# Nephrology Nurses’ Nutritional Competence in Chronic Kidney Disease Care: A Qualitative Study

**DOI:** 10.3390/nursrep16060187

**Published:** 2026-05-28

**Authors:** Sofia Matteucci, Gaetano Ferrara, Giovanni Cangelosi, Ciro Pozzuoli, Sara Morales Palomares, Pasquale Di Fronzo, Anna Grimaldi, Angela Durante, Marco Sguanci, Stefano Mancin, on behalf of the Italian Society of Nephrology Nurses (SIAN) Research Group

**Affiliations:** 1 Scientific Institute for Research, Hospitalization and Healthcare (IRCCS), Humanitas Research Hospital, Via Manzoni 56, 20089 Rozzano, Italy; sofia.matteucci@humanitas.it; 2 Italian Society of Nephrology Nurses (SIAN), Via Capotesta 1/30, 07026 Olbia, Italy; gaetano.ferrara@students.uniroma2.eu (G.F.); pasquale.difronzo@unipr.it (P.D.F.); anna.grimaldi92a@gmail.com (A.G.); sguancim@gmail.com (M.S.); stefano.mancin@humanitas.it (S.M.); 3 Department of Biomedicine and Prevention, University of Rome Tor Vergata, Viale Montpellier 1, 00128 Rome, Italy; ciro.pozzuoli@students.uniroma2.eu; 4 Experimental Medicine and “Stefania Scuri” Public Health Unit, School of Pharmacy, University of Camerino, Via Madonna Delle Carceri 9, 62032 Camerino, Italy; 5 Department of Pharmacy, Health and Nutritional Sciences (DFSSN), University of Calabria, 87036 Rende, Italy; sara.morales@unical.it; 6 Department of Medicine and Surgery, University of Parma, Via Università 12, 43121 Parma, Italy; 7 Tuscany Foundation “G. Monasterio”, Via Giuseppe Moruzzi 1, 56124 Pisa, Italy; adurante@ftgm.it; 8 Health Science Interdisciplinary Center, Scuola Superiore Sant’Anna, Piazza Martiri Della Libertà 33, 56127 Pisa, Italy

**Keywords:** chronic kidney disease, nephrology nursing, nutrition therapy, nursing education, qualitative research, Italy

## Abstract

**Background/Objectives:** Nutritional management is a core component of care for patients with chronic kidney disease (CKD), and nephrology nurses play a key role in education and clinical monitoring. However, how nurses develop and enact nutritional competence in daily practice remains insufficiently explored. This study aimed to explore nephrology nurses’ perceptions and experiences of nutritional management in CKD care. **Methods:** A qualitative descriptive study was conducted through semi-structured interviews with 22 nephrology nurses. Data were analyzed using thematic analysis according to Braun and Clarke. Methodological rigor was ensured following trustworthiness criteria, and reporting adhered to the Consolidated Criteria for Reporting Qualitative Research (COREQ) guidelines. **Results:** The thematic analysis of the interviews identified six main themes: (1) Professional identity and nutritional competence, largely developed through clinical experience rather than structured education. (2) Interprofessional collaboration, perceived as essential but inconsistently implemented. (3) Nutritional education in practice, embedded in daily care and tailored to individual needs. (4) Experiential learning through self-directed nutrition updating. (5) Patient-related challenges, including adherence issues, generational differences, and cultural/educational barriers. (6) Nutritional assessment and decision-making, grounded in routine clinical monitoring and personalized judgment. Participants also highlighted the potential of decision-support tools to enhance personalized nutritional management. **Conclusions:** Strengthening structured nutritional training, improving interprofessional integration, and implementing shared protocols may enhance the consistency, quality, and safety of nutritional care for patients with CKD, supporting more effective translation of evidence into clinical practice.

## 1. Introduction

Chronic kidney disease (CKD) represents a major global public health problem, with an estimated prevalence of approximately 674 million people in 2021, ranking among the leading causes of death and disability worldwide [1]. It has a significant impact on healthcare systems and patients’ quality of life, requiring an integrated and multidimensional approach to care [2]. Among the main complications of CKD, protein–energy malnutrition is a frequent but often underdiagnosed condition [3,4]. Its prevalence varies widely depending on the population studied and the assessment methods used, and it has been estimated that there are between 25% and 40% among hemodialysis patients in Italy [5,6]. This condition is associated with worse clinical outcomes, including increased complications, prolonged hospital stays, and higher mortality rates [7,8]. Nutritional management therefore represents a cornerstone in the care of patients with CKD, as an appropriate dietary intervention can help slow disease progression, reduce complications, and improve overall clinical outcomes [9]. According to the KDOQI Clinical Practice Guideline for Nutrition in CKD, nutritional care should include individualized medical nutrition therapy, nutritional assessment, and dietary counseling tailored to the stage of CKD, dialysis status, comorbidities, biochemical parameters, and patients’ clinical needs. These recommendations emphasize the importance of structured nutrition education and counseling to support dietary adherence, symptom control, and patient self-management across CKD and ESKD care [9]. Early identification of undernutrition risk is equally essential. Recent efforts to develop renal-specific screening tools, such as the Renal Nutrition Screening Tool (R-NST), highlight the complexity of accurately detecting nutritional risk in hospitalized renal patients and the need for structured, clinically sound assessment approaches [10]. However, the feasibility of implementing such tools in routine practice remains challenging, particularly when nursing compliance and integration into daily workflow are suboptimal. Nephrology nurses play a crucial role in promoting dietary adherence, providing nutritional education, and supporting patients’ self-management [11]. As highlighted by Emran and Zolkefli [12], nursing contribution is essential to improving care quality, particularly in delivering effective dietary recommendations to dialysis patients. Nevertheless, empirical evidence suggests that nurses’ nutritional knowledge may only be moderate. For example, Rasheed et al. [13] found that most dialysis nurses demonstrated only fair knowledge regarding nutritional management of renal failure patients, with a smaller proportion achieving high knowledge scores. Such findings raise concerns about the consistency and depth of nutritional preparation among nephrology nurses. Nonetheless, several qualitative studies have shown that the increasing workload, limited resources, and complexity of patients’ needs can lead to occupational stress, emotional exhaustion, and professional frustration among nurses [14,15]. Beyond knowledge levels, qualitative research has emphasized the experiential dimension of nurses’ roles in dietary management. Sulistyaningsih et al. [16], using a phenomenological approach, identified themes related to intensive interaction with patients, multiple educational strategies, perceived barriers, and the need for ongoing professional development in supporting dietary and fluid adherence among hemodialysis patients. These findings suggest that nutritional care is not merely a technical task, but a complex relational and contextual process shaped by workload, communication patterns, and professional confidence. However, evidence on nephrology nurses’ nutritional competence remains limited and inconsistent. These gaps have been linked to heterogeneous educational programs, the lack of standardized competency frameworks, and the insufficient integration of nutritional content into nursing curricula [17,18]. This educational shortfall can reduce the effectiveness of nutritional care and create uncertainty in clinical practice, with potential repercussions on patient outcomes. Beyond technical and clinical aspects, recent literature has emphasized the importance of exploring nurses’ lived experiences and perceptions to understand how they assign meaning to their professional role and caring relationships in nephrology [19]. Such perspectives offer insight into the phenomenological dimension of nursing, revealing the emotional, ethical, and relational components that shape professional practice. For example, Fouché et al. [20] found that caring for patients in terminal stages or following dialysis withdrawal can elicit strong emotional responses, underscoring the need for appropriate organizational and reflective support strategies. However, while existing studies have examined nurses’ knowledge levels or their experiences in delivering dietary education, limited evidence explores how nephrology nurses conceptualize and enact their nutritional competence within the broader context of CKD care, particularly from a qualitative and practice-oriented perspective.

### Aim and Research Question

The aim of the study was to explore nephrology nurses’ perceptions and nutritional competencies in the care of patients with CKD, focusing on how nurses experience their professional role and manage nutritional care in everyday clinical practice.

This study was guided by the following research question:

How do nephrology nurses perceive and experience their nutritional competence in CKD care in everyday clinical practice?

## 2. Materials and Methods

### 2.1. Study Design

This study adopted a qualitative descriptive design [21]. This approach was selected to provide a comprehensive and low-inference account of nephrology nurses’ descriptions regarding nutritional care for patients with CKD. This design is particularly appropriate when the aim is to explore professional practices and competencies in healthcare settings while remaining close to participants’ accounts and avoiding the imposition of strong theoretical interpretations.

### 2.2. Methodological Framework

Thematic analysis as described by Braun and Clarke [22] was used to analyze the interview transcripts for patterns of meaning. This process involved six iterative and reflective phases: (1) familiarization with the data, (2) generation of initial codes, (3) searching for themes, (4) reviewing themes, (5) defining and naming themes, and (6) producing the report. This process enabled the description and connection of themes to answer the research question. Braun and Clarke described the process as recursive, a constant moving back and forth between steps in the analytical process, coded extracts, and the analysis of the data. This approach was selected for its flexibility and its ability to capture patterns of meaning across participants while allowing a nuanced exploration of individual experiences. The analysis was conducted without any software support.

### 2.3. Participant Recruitment

Participants were nephrology nurses working in Italy, in clinical settings, caring for patients with CKD, including outpatient nephrology clinics, hemodialysis units, and peritoneal dialysis services. Eligibility criteria included being a registered nurse, currently working in a nephrology or dialysis setting, having direct clinical experience with patients with CKD, and being involved, at least in part, in nutritional education, monitoring, or support. Nurses not working in nephrology-related settings or without direct experience in CKD care were excluded. Participants were recruited through the Italian Society of Nephrology Nurses (SIAN). The invitation to participate was disseminated through SIAN’s professional network and official email communications to nurses working in nephrology and dialysis settings. Recruitment was not based on a predefined number of invited centers; rather, it aimed to include nurses from different Italian macro-areas and clinical contexts. A purposive sampling strategy was used [23]. The sample size was not determined a priori through statistical calculation, as this was a qualitative study. Recruitment continued until data saturation was achieved, defined as the point at which additional interviews generated no new relevant information and no new relevant themes emerged [24]. Thus, a total of 22 interviews were conducted.

### 2.4. Data Collection

Data collection was carried out through semi-structured, audio-recorded interviews conducted using Microsoft Teams, a web-based Voice over IP (VoIP) platform (Microsoft Corporation, Redmond, WA, USA; version not applicable; available at: https://www.microsoft.com/en-us/microsoft-teams/group-chat-software; accessed on 9 April 2026), after a participation link had been sent by official email. Each participant was assigned an identification code to ensure pseudonymity. Participants provided verbal consent at the beginning of the interview, which was recorded. The average duration of the interviews was 31 min (range: 30–45 min). The interviews were conducted between October and December 2025 by registered nurses (SM and CP) formally trained in qualitative research methods. Both interviewers had clinical backgrounds relevant to the study topic: (CP) was working in a nephrology setting, while (SM) was part of a clinical nutrition team. No prior personal or professional relationship existed between the interviewers and the participants before the interviews. Participants were informed about the study aims and the interviewers’ professional background before participation. Given the interviewers’ clinical expertise in nephrology and nutrition, potential interviewer-related bias was acknowledged and minimized by using the same semi-structured interview guide across all interviews and by asking questions in a neutral, non-leading manner. Each interview followed a semi-structured interview guide developed by the research group. The guide explored nurses’ approaches to nutritional management in patients with chronic kidney disease, the challenges encountered in providing nutritional care, the influence of professional experience and education on nutritional practices, and the perceived impact of the work context (e.g., team support and available resources) on nutritional management. The interviews allowed participants to freely share their experiences and describe nutritional care for patients with CKD, including perceived competencies, confidence in nutrition-related tasks, challenges encountered in everyday clinical practice, and emotional and organizational factors influencing nutritional management. Consistency across interviews was maintained by using the same interview guide for all participants, while the semi-structured nature of the interviews allowed flexibility, enabling researchers to follow the order of questions according to the flow of the interview and allowing participants to discuss aspects most relevant to their personal experiences [25].

### 2.5. Data Analysis

All interviews were transcribed verbatim using the automatic transcription function of Microsoft Teams (Microsoft Corporation, Redmond, WA, USA; version not applicable; available at: https://www.microsoft.com/en-us/microsoft-teams/group-chat-software; accessed on 9 April 2026) and then manually reviewed to correct any errors. To gain in-depth familiarity with the interview corpus, two investigators listened to the audio recordings while reading and rereading the interview transcripts. The analysis followed an inductive approach, as codes, subthemes, and themes were derived from the interview data rather than being predefined according to an existing theoretical framework. Two investigators (SM and CP) independently coded the transcripts to identify preliminary units of meaning and initial codes. Discrepancies were discussed collegially, and in cases of persistent disagreement, a third member of the research team (PDF) was involved. The final version of the thematic structure was shared and validated with the entire research group. Themes and sub-themes were generated, based on consensus among researchers. In line with the reflective orientation of this approach, the development of themes was understood as an interpretative and recursive process shaped by the analytical decisions of the researchers, rather than as a purely mechanical procedure [26].

#### Development of Themes and Subthemes

In this study, themes represented broader patterns of meaning across participants’ accounts, while subthemes captured more specific dimensions within each theme. Themes were developed by grouping related codes according to their conceptual similarity and relevance to the research question. Themes were separated by comparing the central meaning of each group of codes and assessing whether they reflected distinct aspects of nurses’ nutritional competence and practice. The final thematic structure was discussed and refined within the research team to ensure internal coherence within themes and clear distinction between themes.

### 2.6. Trustworthiness

We employed the trustworthiness criteria proposed by Lincoln and Guba [27], following the practical guidance provided by Korstjens & Moser [28]. Credibility was ensured by providing accurate reports of the entire research process and reaching consensus within the research group on the interpretation of the data. This process involved the complete transcription of the recorded interviews to ensure transparency and consistency; individual analysis by independent researchers, with an additional researcher involved in cases of disagreement, and sharing and discussion of emerging themes with the research team to reach a shared definition. Member validation was not conducted, as returning transcripts or final findings to participants was not included in the study design. The analysis was based on verbatim interview transcripts and on the interpretative work of the research team; therefore, credibility was further supported through independent coding, collegial discussion of discrepancies, and agreement on the final thematic structure. Transferability was ensured by providing comprehensive descriptions of the study design, participants, context, sampling, data collection, and analysis. Dependability was ensured by critically examining the quality of each stage of data collection and analysis. Confirmability was supported through documentation of analytic decisions throughout the research process. The study was conducted in accordance with the Enhancing the Quality and Transparency Of health Research (EQUATOR) Network guidelines for reporting research and is reported following the 32-item Consolidated Criteria for Reporting Qualitative Research (COREQ) checklist [29]. The completed COREQ checklist is available as Supplementary File S1.

### 2.7. Ethical Considerations

The study was conducted in accordance with the Declaration of Helsinki. The protocol was reviewed and approved by the Scientific Committee of the SIAN under approval number SIAN.RIC 01/2025, dated 30 April 2025. All participants provided a recorded verbal consent at the beginning of the interview, prior to the start of data collection. Furthermore, the study was conducted with respect for participants’ autonomy, confidentiality, and anonymity. Each interview was assigned a unique identification code and stored in a secure database to ensure participant pseudonymity. Audio recordings were securely stored and were not disclosed outside the research team. Recordings were permanently deleted after the completion of the analysis, in accordance with applicable data protection regulations.

## 3. Results

A total of 22 nurses working in nephrology and dialysis settings were interviewed. The average duration of the interviews was approximately 31 min (range: 30–45 min). Participants were recruited from different Italian macro-areas (North, Center, South/Islands) and from various types of healthcare facilities [HUB, SPOKE, Limited Care Assistance Center (LCAC), and private/accredited centers]. The sample was predominantly female. Detailed participant characteristics are reported in Table 1.

**Figure 1:**
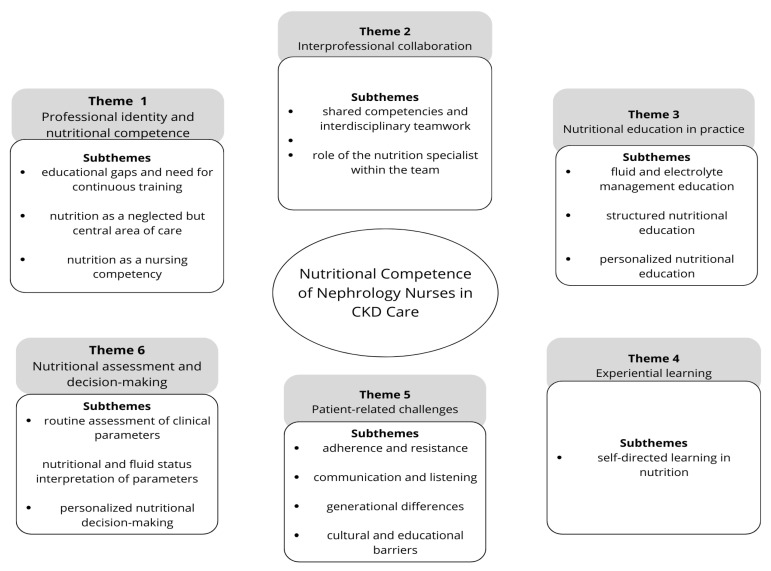
Themes and subthemes emerging from the thematic analysis.

Thematic analysis led to the identification of six main themes, each articulated into subthemes: (1) Professional identity and nutritional competence. (2) Interprofessional collaboration. (3) Nutritional education in practice. (4) Experiential learning. (5) Patient-related challenges. (6) Nutritional assessment and decision-making. Although Theme 1 and 4 are conceptually related, they represent distinct dimensions of nurses’ nutritional competence. Theme 1 refers to how nurses perceive nutritional competence as part of their professional identity and role, whereas Theme 4 focuses on how this competence is developed over time through clinical experience and self-directed learning. The themes and subthemes that emerged from the thematic analysis are illustrated in Figure 1.

### 3.1. Theme 1: Professional Identity and Nutritional Competence

Nurses reported educational gaps at the time of entering dialysis practice and expressed the need for further training, recognizing nursing competence as a central element of care.

#### 3.1.1. Educational Gaps and Need for Continuous Training

Participants described limited initial education in nutrition:

*“So yes, we more or less give advice, but I don’t feel able to go into much depth because my knowledge is limited, unfortunately… These are things you learn on the job. No one teaches you, because we never really had training in the nutritional field. It’s a very neglected area. There is a lack of information, a lack of everything.”*

(INT_01)

#### 3.1.2. Nutrition as a Neglected but Central Area of Care

Nutrition was recognized as part of the treatment itself rather than merely additional advice:

*“Advising a healthy diet to a dialysis patient is, in my opinion, 50% of the treatment. A well-nourished patient is less inflamed, avoids anorexia, maintains stable weight, has fewer nutritional problems, manages food properly, and ultimately responds better to treatment.”*

(INT_06)

However, ambivalent attitudes emerged regarding the assumption of additional responsibilities and motivation for professional development:

*“There is little willingness to update or grow in competencies, because ‘they are extra responsibilities,’ ‘others will take care of it,’ ‘it’s not my role.’”*

(INT_22)

#### 3.1.3. Nutrition as a Nursing Competency

Many nurses acknowledged their role in progressively influencing patients’ dietary habits:

*“The only one who can influence their habits is the nurse, who sees them three times a week and can gradually understand what to suggest and how.”*

(INT_06)

### 3.2. Theme 2: Interprofessional Collaboration

Nutritional care was perceived as multidisciplinary, although collaboration was uneven.

#### 3.2.1. Shared Competencies and Interdisciplinary Teamwork

Nephrology nursing competencies require collaboration and sharing, yet in some settings team-based work was not feasible:

*“I cannot carry out these activities alone… A nurse acting alone does not make much sense. A shared approach is necessary, where everyone works in the same way, because only then can the pathway function.”*

(INT_11)

Some participants reported limited direct involvement of dietitians in their clinical setting, particularly in relation to patient education and staff training:

*“I have never seen a dietitian in the ward explaining to patients how they should eat or providing training to us.”*

(INT_15)

#### 3.2.2. Role of the Nutrition Specialist Within the Team

The presence of a dietitian was considered an added value:

*“If at least once a year we could meet with a dietitian who guides us, we would definitely do it, because in my opinion it would be enriching.”*

(INT_10)

Other professionals could also support adherence and understanding:

*“It would be appropriate to introduce a figure such as a psychologist, for example. These professionals can help patients adopt a different approach to dialysis, and therefore perhaps a more appropriate approach to what they eat.”*

(INT_08)

### 3.3. Theme 3: Nutritional Education in Practice

Structured and personalized education on diet and hydration was perceived as necessary.

#### 3.3.1. Fluid and Electrolyte Management Education

Nurses provided practical nutritional advice, especially during the initial phase of dialysis:

*“I provide some advice, especially to patients starting dialysis, for example suggesting smaller measuring cups or glasses, double boiling vegetables, and choosing pasta instead of rice, which absorbs more water.”*

(INT_22)

#### 3.3.2. Structured Nutritional Education

Nutritional education did not occur as a scheduled intervention but emerged during routine care, integrating dietary conversations into standard clinical activities:

*“We do it directly at the patient’s bedside. Every time we connect them to the machine, we ask about their daily diet and provide guidance on what foods they can eat and how much fluid they are allowed.”*

(INT_02)

#### 3.3.3. Personalized Nutritional Education

Nutritional approaches varied according to patient characteristics, particularly gender and relational complexity:

*“Our approach is very human, because for patients it is already difficult to accept dialysis. In my experience, it is often even more complex for men, who tend to eat and drink more than women. That’s why we have to proceed gradually: you can’t immediately say ‘you must not eat this’ or ‘you must not drink that,’ but you need to help them understand, guiding them calmly and serenely, and sometimes explaining small practical ‘tricks.’”*

(INT_05)

### 3.4. Theme 4: Experiential Learning

Nutritional competencies were mainly developed through direct clinical experience.

#### Self-Directed Learning in Nutrition

Knowledge was acquired through experience:

*“My knowledge in nutrition comes from my professional experience.”*

(INT_05)

Self-directed updating was often triggered by perceived knowledge gaps:

*“My interest started when patients asked, ‘Can I eat this?’ I didn’t know how to answer at that moment, so I realized it was a gap and tried to update myself.”*

(INT_11)

### 3.5. Theme 5: Patient-Related Challenges

Difficulties primarily concerned adherence, generational differences, and ethnic-cultural variables.

#### 3.5.1. Adherence and Resistance

Patients with chronic conditions were often perceived as difficult to manage and sometimes reluctant to receive advice:

*“It’s a complex issue and often underestimated. It’s complex especially because chronic patients, even psychologically, tend to self-manage and are convinced they know what is good or bad for them. For this reason, they are often resistant to advice.”*

(INT_06)

#### 3.5.2. Communication and Listening

Continuity of care and relational engagement promoted adherence and motivation:

*“Taking care of them continuously, maybe even with an extra phone call. I have seen that this always pays off. The fact that we care and establish a confidential relationship is rewarding, because I see they follow us much more and feel gratified for their efforts.”*

(INT_16)

#### 3.5.3. Generational Differences

Approaches varied according to patient age:

*“Most of them listen little, especially the younger ones.”*

(INT_02)

However, contrasting views emerged:

*“Sometimes there is a lack of compliance, also because we have a population with an average age around 80. Making certain things understood is difficult… With younger patients it is obviously easier to work; with an eighty- or ninety-year-old it is not, so we have to adjust our goals.”*

(INT_10)

#### 3.5.4. Cultural and Educational Barriers

Providing guidance was sometimes difficult due to ethnic-cultural differences:

*“We now have patients from different ethnic backgrounds, each with different cultural habits, and in some cases it is really difficult to guide them toward proper nutrition, also because we lack knowledge about other cultures.”*

(INT_09)

*“If you give the wrong indication, for example suggesting steak, but for that person, based on their culture and habits, steak is not appropriate, then you need to propose an alternative that is more consistent with their reality.”*

(INT_19)

*“I tried with some patients, because they kept gaining weight, probably due to excessive fluid intake, as they were unable to distinguish water from broth or other beverages.”*

(INT_07)

### 3.6. Theme 6: Nutritional Assessment and Decision-Making

Nutritional assessment was integrated into clinical practice through routine monitoring and the use of validated tools.

#### 3.6.1. Routine Assessment of Clinical Parameters

The monitoring of clinical parameters was recognized as the foundation of nursing care for dialysis patients. Participants particularly emphasized body weight as a daily indicator used to support the assessment of fluid balance:

*“Weight is assessed every day they come in.”*

(INT_05)

Regular blood tests were also described as part of routine monitoring to support the evaluation of nutritional status:

*“Every month, we perform blood tests to check albumin and understand whether the patient is eating properly. We also check phosphorus and potassium, sometimes more than once a month.”*

(INT_13)

#### 3.6.2. Nutritional and Fluid Status Interpretation of Parameters

Participants emphasized the importance of assessment tools:

*“We assess the risk of malnutrition as a team using the MUST scale.”*

(INT_09)

*“Hospitalized patients undergo nutritional status assessment scales to detect their nutritional condition.”*

(INT_12)

#### 3.6.3. Personalized Nutritional Decision-Making

Nutrition was modulated according to disease stage:

*“It starts from the lower levels, from level one and two up to level five, which then leads to the dialysis pathway, because before entering dialysis patients are put on protein-free or reduced-sodium and reduced-protein diets.”*

(INT_12)

## 4. Discussion

This study explored nephrology nurses’ perceptions and experiences regarding the nutritional management of patients with CKD. Overall, nutrition was recognized as a central component of care; however, it is delivered within a context characterized by educational gaps, strong reliance on experiential learning, continuous relational negotiation, and multidisciplinary integration that is not always structured [30,31]. In this context, the nursing role emerged as particularly relevant in its educational dimension, while participants also emphasized the need for more structured and adequate nutritional training for nurses. Rather than being a formally defined clinical competence, nutritional competence emerged as a situated practice, constructed within daily care and shaped by patient interaction, organizational dynamics, and contextual constraints [32]. Participants described nutritional competence as progressively developed after entering the nephrology setting rather than acquired during undergraduate education. This finding is consistent with existing literature: quantitative studies have documented specific gaps in nutritional knowledge among hemodialysis nurses, particularly in phosphorus management [33], knowledge levels sometimes comparable to those of patients [34], and more recently, a systematic review confirming only moderate levels of nutritional preparation [35]. Furthermore, a national survey by Gazineo et al. [31] identified specific gaps in the nutritional domain despite high perceived levels of decision-making and communication competencies. Collectively, this evidence supports the need for more structured, homogeneous, and institutionally recognized educational pathways. These findings suggest that nutritional competence is not merely an educational deficit but reflects a dynamic balance between clinical responsibility, professional autonomy, and organizational limitations. The experiential learning described by participants aligns with the framework outlined by Parozzi et al. [30], which highlights how nephrology competencies develop through a combination of structured education and professional experience in the absence of internationally standardized training models. Similarly, the framework proposed by Pegoraro et al. [36] conceptualizes nephrology competence as the dynamic integration of knowledge, skills, and attitudes (“knowing, doing, being”), emphasizing the progressive and reflective nature of professional maturation. From this perspective, nutritional competence can be interpreted as a situated competence constructed through the interaction between formal knowledge and tacit experiential learning. Beyond the cognitive dimension, findings revealed a significant identity tension. On one hand, nurses recognized nutrition as an integral part of treatment; on the other, ambivalence emerged regarding role boundaries and perceived responsibility. This dynamic is consistent with Latzourakis et al. [37], who describe nephrology nursing as encompassing technical, educational, coordinative, and emotional roles influenced by organizational and educational contexts. Nutritional competence appears to occupy this space of professional negotiation, where nurses continuously redefine their contribution within the multidisciplinary team. Nutritional management also emerged as a deep relational practice. Dietary education was described as gradual, negotiated, and personalized, adapted to patients’ age, clinical condition, cultural habits, and emotional readiness. This relational dimension is consistent with qualitative studies portraying dietary education as a flexible and adaptive process sustained over time through differentiated strategies [12,21]. Moreover, the scoping review by Mancin et al. [38] highlights the crucial role of relational skills such as active listening, empathy, and shared decision-making in promoting adherence and therapeutic autonomy. In this sense, nutritional competence encompasses not only technical but also relational and cultural dimensions. Importantly, this relational component does not oppose clinical reasoning; rather, it enacts it, as nutritional decisions arise from the integration of objective data and therapeutic negotiation. Findings also highlighted critical issues in interdisciplinary collaboration, particularly the inconsistent presence of dietitians in clinical settings. This aligns with Papier et al. [39], who identified organizational barriers, such as the absence of shared protocols and perceptual divergences between professional levels, as obstacles to effective nutritional management. In such contexts, nurses may assume a compensatory role, translating general prescriptions into practical daily guidance and bridging organizational gaps. Another key element concerned the evaluative and decisional dimension of nutritional competence. Nurses described the systematic integration of objective parameters, such as interdialytic weight gain, biochemical markers (albumin, phosphorus, potassium), and standardized tools to detect the risk of malnutrition, such as the MUST, into nutritional management. This integration reflects applied clinical reasoning, whereby nurses interpret clinical data to tailor educational interventions and modulate care strategies. This dimension is consistent with the core and advanced competencies described by Andreoli et al. [32] and aligns with findings by Gazineo et al. [31], which report high perceived decision-making competence among nephrology nurses. Nutrition thus emerges as a privileged domain for the exercise of nursing clinical autonomy. In parallel, recent evidence suggests that strengthening structured nursing competencies in CKD care may translate into measurable clinical outcomes. A systematic review by Tai et al. [40] demonstrated that educational interventions for nurses caring for patients with CKD significantly improved knowledge, clinical performance, decision-making, and nurse–patient interaction, with sustained effects over time. At the interventional level, Shi et al. [41] demonstrated that a nurse-led intensive educational program significantly improved hyperphosphatemia control, while Wong et al. [42] showed that nurse-led disease management models were associated with improvements in dietary adherence and quality of life. Nutritional competence therefore represents not only a professional dimension but also a potential determinant of clinical stability, patient safety, and quality of care. Unlike studies that have explored the nursing role in CKD more broadly, the present study specifically highlights nutrition as a privileged domain for the exercise of clinical autonomy, underscoring its multidimensional and situated nature. Overall, findings suggest that nutritional competence represents a pivotal node within nephrology practice, positioned at the intersection of clinical autonomy, therapeutic relationship, and organizational governance, and therefore requiring formal recognition and systemic support.

### 4.1. Limitations and Strengths

The study has several limitations. As it was conducted within a specific national context, the findings may not be fully transferable to other healthcare contexts and systems. Furthermore, as a qualitative interview-based study, the reported descriptions reflect subjective experiences and may have been influenced by social desirability bias. In addition, self-selection bias cannot be excluded, as nurses who voluntarily agreed to participate may have had a greater interest in nutritional care or professional development than those who did not participate. Interviewer-related bias should also be considered, as participants’ responses may have been influenced by the presence of the interviewer and by the interaction established during the interview. An additional limitation concerns the potential heterogeneity of participants’ organizational contexts, which may have shaped the experiences described. Data on the actual availability of dietitians or nutritionists within participants’ practice settings or healthcare teams were not systematically collected. Therefore, it was not possible to determine whether nutrition professionals were formally available or responsible for patient nutrition education across the included settings. Information on participants’ specialized nephrology training was not collected as a separate variable. Therefore, the potential influence of prior nephrology-specific education on nurses’ nutritional competencies and knowledge could not be explored. Moreover, although nurses were recruited from different nephrology settings, most participants were working in hemodialysis units; this prevalence may have influenced the findings by emphasizing experiences more closely related to the hemodialysis context, potentially limiting the representation of other nephrology care settings. Among its strengths, the qualitative descriptive approach allowed for an in-depth exploration of the identity-related, relational, and organizational dynamics associated with nutritional competence. The inclusion of nurses from diverse clinical settings enabled the capture of multiple perspectives, contributing to a comprehensive and realistic portrayal of nutritional practice in nephrology. The study therefore provides an original contribution to literature and offers a foundation for future research aimed at developing more structured educational and organizational models within nephrology nursing.

### 4.2. Implications for Practice

This study highlights that nutritional competence in nephrology nursing extends beyond the acquisition of technical knowledge and should be understood as a multifaceted professional capability shaped through clinical practice. In the context of CKD, nutritional care represents a clinically relevant domain in which assessment skills, clinical reasoning, relational abilities, and decision-making processes intersect. However, despite its relevance within CKD management, nutritional competence does not appear to be consistently formalized within educational pathways or organizational frameworks, which may contribute to variability across settings and reliance on individual experience. In line with this, evidence suggests that exposure to nutrition courses and structured continuing education and training are associated with higher levels of nutritional nursing competence, supporting the value of formalized training strategies [43]. At the organizational level, the complexity of nutritional management in CKD further supports the importance of interdisciplinary collaboration. Renal dietitians have a central role in delivering medical nutrition therapy and supporting comprehensive kidney care, yet access to specialized renal dietetic expertise remains uneven worldwide, indicating that interprofessional models may not be consistently implemented across contexts [44]. Finally, variability in practice may also be mitigated through the integration of standardized assessment approaches; recent evidence shows that the MUST demonstrates high diagnostic accuracy in identifying malnutrition risk in hospitalized adults, reinforcing its relevance as a structured component of nutritional assessment when combined with clinical judgment [45].

## 5. Conclusions

This study underscores the relevance of nutritional competence as a core dimension of nephrology nursing practice. Although nutritional care is integral to CKD management, its translation into structured educational pathways and organizational routines remains inconsistent across settings. Developing standardized training and competency frameworks, alongside stronger interdisciplinary collaboration and shared protocols, may improve the consistency and safety of nutritional care and support the ongoing professional development of nephrology nurses. These findings provide actionable insights for structuring nutritional training pathways and improving interdisciplinary integration in nephrology nursing practice.

## Figures and Tables

**Table 1:** Participant characteristics (N = 22).

Variables	Values, N (%)
Gender	
Female	13 (59.1)
Male	9 (40.9)
Region	
North	11 (50.0)
Center	4 (18.2)
South/Islands	7 (31.8)
Education	
Bachelor’s Degree	11 (50.0)
Postgraduate	11 (50.0)
Age (years) M (SD), range	44.0 (10.8), 25–65
Nephrology nursing experience (years) M (SD), range	14.2 (11.5), 2–44
Ward	
Hemodialysis	17 (77.3)
Peritoneal dialysis	1 (4.5)
Nephrology ward	1 (4.5)
Transplant area	1 (4.5)
Other	2 (9.1)
Center type	
HUB (III level)	9 (40.9)
SPOKE (II level)	2 (9.1)
LCAC (I level)	5 (22.7)
Private/accredited (I level)	6 (27.3)

Legend. Hub (III Level): Tertiary Referral Center; Spoke (II Level): Secondary-Level Center; LCAC (I Level): Limited Care Assistance Center (Low-Intensity Care or Outpatient Service); N: Number; %: Percentage; M: Mean; SD: Standard Deviation.

## Data Availability

The data presented in this study are available on reasonable request from the corresponding author. The data are not publicly available due to ethical and privacy restrictions.

## Supplementary Materials

The following supporting information can be downloaded at https://www.mdpi.com/article/10.3390/nursrep16060187/s1, Supplementary File S1: COREQ 32-item checklist.

## Public Involvement Statement

No public involvement in any aspect of this research.

## Guidelines and Standards Statement

This manuscript was drafted in accordance with the Consolidated Criteria for Reporting Qualitative Research (COREQ) guideline for qualitative research and follows the EQUATOR Network recommendations [29].

## Abbreviations

The following abbreviations are used in this manuscript:

LCAC	Limited Care Assistance Center (I level)
CKD	Chronic Kidney Disease
COREQ	Consolidated Criteria for Reporting Qualitative Research
EQUATOR	Enhancing the Quality and Transparency Of health Research
HUB	Tertiary Referral Center (III level)
INT	Interview
MUST	Malnutrition Universal Screening Tool
R-NST	Renal Nutrition Screening Tool
SIAN	Italian Society of Nephrology Nurses
SPOKE	Secondary-Level Center (II level)
VoIP	Voice over IP

## Appendix A

Italian Society of Nephrology Nurses (SIAN) Board of Directors (contributed as authors of the manuscript):

1. Domenica Gazineo: Italian Society of Nephrology Nurses (SIAN) Board of Directors, Via Capotesta 1/30, 07026 Olbia, Italy; Governo Clinico e Qualità, IRCCS Azienda, Ospedaliero-Universitaria di Bologna, Bologna, Italy; domenica.gazineo3@unibo.it;
2. Addolorata Palmisano: Italian Society of Nephrology Nurses (SIAN) Board of Directors, Via Capotesta 1/30, 07026 Olbia, Italy; dorianapalmisano77@gmail.com;
3. Alessandro Pizzo: Italian Society of Nephrology Nurses (SIAN) Board of Directors, Via Capotesta 1/30, 07026 Olbia, Italy; pizzoalexxandro@gmail.com;
4. Silvia Cappelletti: Italian Society of Nephrology Nurses (SIAN) Board of Directors, Via Capotesta 1/30, 07026 Olbia, Italy; silviacappelletti74@outlook.com;
5. Angela Greco: Italian Society of Nephrology Nurses (SIAN) Board of Directors, Via Capotesta 1/30, 07026 Olbia, Italy; angelagreco1967@gmail.com;
6. Francesco Barci: Italian Society of Nephrology Nurses (SIAN) Board of Directors, Via Capotesta 1/30, 07026 Olbia, Italy; francescobarci81@gmail.com.
