# Supplementary material for: Nephrology Nurses’ Nutritional Competence in Chronic Kidney Disease Care: A Qualitative Study
Source: Nurs Rep. 2026 May 28;16(6):187. doi: 10.3390/nursrep16060187 (PMC13304488; doi:10.3390/nursrep16060187)
Supplement: Supplementary file 1 [file nursrep-16-00187-s001.zip › nursrep-4281753-supplementary.pdf]

Supplementary file S1 (COREQ): 32-item checklist

No	Item	Guide questions/description	Response	Page / Justification
**Domain 1: Research team and reflexivity Personal Characteristics**				
1	Interviewer/facilitator	Which author/s conducted the interview or focus group?	Interviews were conducted by S.M. and C.P.	p. 3-5
2	Credentials	What were the researcher's credentials? E.g. PhD, MD	Both interviewers held a Bachelor's degree in Nursing (RN).	p. 3-5
3	Occupation	What was their occupation at the time of the study?	At the time of the study, one interviewer was a registered nurse working in a nephrology setting, and the other was a registered nurse in a clinical nutrition team.	p. 3-5
4	Gender	Was the researcher male or female?	The interviews were conducted by one female and one male researcher.	p. 3-5
5	Experience and training	What experience or training did the researcher have?	The interviewers had prior experience in conducting interviews. Methodological rigor was ensured through team discussions and collaborative data analysis.	p. 3-5
**Relationship with participants**				
6	Relationship established	Was a relationship established prior to study commencement?	No prior relationship was established before study commencement.	p. 3-5
7	Participant knowledge of the interviewer	What did the participants know about the researcher? e.g. personal goals, reasons for doing the research	Participants were informed about the research aims, the interviewers' professional background, and the purpose of the study prior to participation.	p. 3-5
8	Interviewer characteristics	What characteristics were reported about the interviewer/facilitator? e.g.	Interviews were conducted by two registered nurses with clinical experience in nephrology. One interviewer was undertaking postgraduate	p. 6-7

		Bias, assumptions, reasons and interests in the research topic	studies in Human Nutrition Sciences, reflecting a specific academic interest in nutritional care. Reflexivity was addressed through regular team discussions and critical reflection on potential assumptions, professional preconceptions, and possible sources of bias.	
**Domain 2: study design**<br>**Theoretical framework**				
9	Methodological orientation and Theory	What methodological orientation was stated to underpin the study? e.g. grounded theory, discourse analysis, ethnography, phenomenology, content analysis	Qualitative descriptive design using thematic analysis according to Braun and Clarke (2006).	p. 3-4
**Participant selection**				
10	Sampling	How were participants selected? e.g. purposive, convenience, consecutive, snowball	Purposive sampling with maximum variation was used to ensure diversity in professional experience, clinical setting, and years of practice.	p. 4
11	Method of approach	How were participants approached? e.g. face-to-face, telephone, mail, email	Participants were approached through the Italian Society of Nephrology Nurses (SIAN) via email communication.	p. 4
12	Sample size	How many participants were in the study?	Twenty-two nephrology nurses participated in the study.	p. 4-5
13	Non-participation	How many people refused to participate or dropped out? Reasons?	No participants withdrew after providing consent. Information on initial refusals was not systematically recorded.	p. 4-5
**Setting**				
14	Setting of data collection	Where was the data collected? e.g. home, clinic, workplace	Data were collected remotely via an institutional Voice over IP (VoIP) system. Interviews were conducted online according to participants' availability and preferences.	p. 4-5

15	Presence of non-participants	Was anyone else present besides the participants and researchers?	No. Only participants and researchers were present.	p. 4-5
16	Description of sample	What are the important characteristics of the sample? e.g. demographic data, date	The sample included 22 nephrology nurses from different Italian macro-areas (North, Centre, South/Islands) working in dialysis and nephrology settings (HUB, SPOKE, CAL, and private/accredited centers). The majority were female (59.1%), with a mean age of 44 years and a mean professional experience of 14.2 years. Educational levels included Bachelor's and postgraduate degrees.	p. 6–7 (Table 1)
		**Data collection**		
17	Interview guide	Were questions, prompts, guides provided by the authors? Was it pilot tested?	A semi-structured interview guide consisting of four open-ended questions was developed by the research team based on the study aims and relevant literature. The guide was refined through team discussion and was not formally pilot tested.	p. 4-5
18	Repeat interviews	Were repeat interviews carried out? If yes, how many?	No. Repeat interviews were not conducted.	Repeat interviews were not planned, as the study was designed to collect participants' accounts through a single semi-structured interview. Data collection continued until thematic saturation was achieved, with no need for follow-up interviews.
19	Audio/visual recording	Did the research use audio or visual recording to collect the data?	Yes. Before commencing the interviews, verbal informed consent was obtained from all participants. Audio recording began only after participants explicitly provided consent.	p. 5
20	Field notes	Were field notes made during and/or after the interview or focus group?	No. Formal field notes were not taken during or after the interviews.	Interviews were conducted online and fully audio-recorded. Since the analysis focused on the verbal content of participants' narratives, verbatim transcripts constituted the primary data

				source, and observational field notes were not considered necessary.
21	Duration	What was the duration of the interviews or focus group?	Interviews lasted approximately 30–45 minutes, with a mean duration of 31 minutes.	p. 5
22	Data saturation	Was data saturation discussed?	Yes. Data collection continued until thematic saturation was achieved, defined as the point at which no new relevant themes emerged from the interviews.	p. 4-5
23	Transcripts returned	Were transcripts returned to participants for comment and/or correction?	No. Transcripts were not returned to participants for comment or correction.	Returning transcripts to participants was not included in the study design. The analysis was conducted using verbatim interview transcripts as the primary data source.
**Domain 3: analysis and findings**<br>**Data analysis**				
24	Number of data coders	How many data coders coded the data?	Two researchers independently conducted the initial coding of the data using an inductive approach. Codes and themes were subsequently reviewed and discussed within the research team.	p. 4-5
25	Description of the coding tree	Did authors provide a description of the coding tree?	Yes. The thematic framework, including six main themes and related subthemes, is described in the Findings section and illustrated in Figure 1. The development of themes followed an iterative analytic process.	p. 7
26	Derivation of themes	Were themes identified in advance or derived from the data?	Themes were inductively derived from the data following Braun and Clarke's six-phase thematic analysis framework. No themes were predetermined prior to data analysis.	p. 7
27	Software	What software, if applicable, was used to manage the data?	No qualitative data analysis software was used. Coding and thematic analysis were conducted manually by the research team.	p. 5
28	Participant checking	Did participants provide feedback on the findings?	No. Participants did not provide feedback on the final findings (member checking was not conducted).	Participant validation (member checking) was not included in the study design, as the thematic analysis focused

				on the interpretative work of the research team based on verbatim interview transcripts.
**Reporting**				
29	Quotations presented	Were participant quotations presented to illustrate the themes / findings? Was each quotation identified? e.g. participant number	Yes. Participant quotations were presented to illustrate each theme and subtheme. Each quotation was identified using an anonymized interview code (e.g., INT_01, INT_06), ensuring confidentiality while allowing traceability of perspectives.	p. 8-13
30	Data and findings consistent	Was there consistency between the data presented and the findings?	Yes. The findings were directly grounded in participants' narratives. Each major theme and subtheme was supported by illustrative quotations, ensuring alignment between the raw data and the interpretative claims.	p. 8-13
31	Clarity of major themes	Were major themes clearly presented in the findings?	Yes. Six major themes were clearly identified and explicitly described in the Findings section. Each theme was structured with corresponding subthemes and supported by participant quotations. The thematic framework was also illustrated in Figure 1 to enhance clarity.	p. 8-13
32	Clarity of minor themes	Is there a description of diverse cases or discussion of minor themes?	Yes. Subthemes within each major theme reflect diverse perspectives and contrasting viewpoints (e.g., generational differences in adherence, ambivalence regarding professional responsibility, variability in interdisciplinary collaboration). Minor or divergent perspectives were integrated within subthemes rather than presented as standalone themes.	p. 8-13
